# Cormorants as a Potentially Important Reservoir and Carrier of Newcastle Disease Virus on the Asian Continent

**DOI:** 10.3389/fvets.2021.648091

**Published:** 2021-06-14

**Authors:** Kobey Karamendin, Aidyn Kydyrmanov

**Affiliations:** ^1^Laboratory of Viral Ecology, Scientific and Production Center of Microbiology and Virology, Almaty, Kazakhstan; ^2^Kazakh National Agrarian University, Almaty, Kazakhstan

**Keywords:** cormorant, Newcastle disease virus, velogenic strain, Asia, reservoir, poultry

## Abstract

Despite numerous disease prevention measures and control programs, Newcastle disease (ND) remains one of the most significant infections in poultry worldwide, especially in developing countries. It is known that wild birds, mainly of the *Anseriformes* order, are the main carrier of lentogenic (non-pathogenic) variants of Newcastle disease virus (NDV) in nature. But the question of the reservoir of velogenic (highly pathogenic) NDV in nature still remains open. In the 1970s, 1990s, and 2000s in North America during epizootics among cormorants, velogenic NDV strains were isolated. It was later concluded that cormorants play an important role in the maintenance and circulation of NDV in North America. New data have been obtained on the circulation of velogenic NDV strains in wild birds in Central Asia: VIIb and XIII genotype strains were isolated from cormorants for the first time in Kazakhstan. Interestingly, outbreaks of NDV registered in poultry in Central and Southern Asia were phylogenetically close to the viruses from cormorants that support the idea that cormorants can serve as the potential reservoir of velogenic NDV in developing countries of Asia. The seasonal migrations of cormorants may contribute to the distribution of the virus throughout Asia but more evidence must be obtained to confirm this hypothesis. There is increasing evidence of the introduction of NDV into the poultry farms from wild nature worldwide. This article continues the discussion on the likelihood of cormorants to serve as a reservoir and carrier of NDV on the Asian continent.

## Introduction

Newcastle disease is a viral disease of wild and domestic birds, causing mass mortality in poultry, mainly in developing countries. It is known that birds of the *Anseriformes* order are the reservoir of lentogenic NDV in nature (1). Thousands of cases of isolation of lentogenic strains from wild birds during monitoring for influenza virus have been described, but the origin and reservoir of velogenic NDV in nature are still unknown (2). From 1975 to 2010 there were several outbreaks of NDV among the local population of cormorants in North America (3). Cases of clinical manifestation of the disease with the isolation of velogenic strains were described in detail. Due to the effective protection system in the poultry industry in North America, outbreaks among poultry have not been reported. Only domestic turkeys were affected in North Dakota (4).

After numerous cases of isolation of velogenic NDV from cormorants in North America, they were considered a reservoir species of NDV in the local avifauna (5). In Eurasia, similar cases of detection of velogenic NDV from cormorants were revealed in Scotland in 1949 (6). In 1974, strains of velogenic NDV were isolated from cormorants in the northern Caspian (7), but mortality among cormorants was not recorded at that time. Then, in 2014 and 2016, mortality was recorded among juvenile cormorants in Central Asia at Lake Alakol (8, 9). Simultaneously with the detection of the velogenic NDV in cormorants, epizootic outbreaks with high mortality in local poultry farms were registered. It was defined that outbreaks in poultry were caused by very similar strains of the highly pathogenic NDV genotype VII (10) that were described in cormorants.

The purpose of this study was to compare the American and Eurasian strains of NDV from cormorants and try to answer the question of what is the likelihood that cormorants are responsible for mass die-offs in developing Asian countries.

## Outbreaks of VELOGENIC NDV Among Cormorants in North America

The first evidence of NDV in cormorants was registered in Quebec, Canada in 1975, when the virus was isolated from 14 of 27 (52%) birds with signs of ocular opacity, partial paralysis, and extreme weakness (11). Then, after a long time, new cases of mortality in cormorants caused by NDV were registered in 1990 and 1992 in Canada and the USA (12). For the first time in North Dakota, an infection of domestic turkeys with NDV was observed in 1992, the origin of which was associated with NDV from cormorants (4). Notably, in addition to cormorants, mortality was simultaneously observed among gulls and pelicans. The analysis of NDV isolates using monoclonal antibodies did not reveal a difference between isolates of 1975, 1990, and 1992, as well as isolates from turkeys (13). An interesting study was carried out between two epizootics in 1990 and 1992: when examining the yolks of eggs from cormorants, gulls and pelicans in 1991, antibodies to NDV were found in 50% of cases, and no mortality was observed among them in 1991 (14). During the epizootics of 1990 and 1992, signs of disease were described in cormorants, which included inability to fly, often with unilateral wing or leg paralysis. Some birds appeared unafraid or unaware of humans, while others tried to escape but were unable to fly. The absence of sick birds with similar signs among gulls was also noted. The clinical signs in pelicans were similar to that in cormorants. One more important observation was that all died cormorants were young-of-the year.

It was noted that isolation of NDV from sick or dead birds was not always possible in North America. Several factors were suggested that may have contributed to the failure to isolate NDV from many of the birds examined. The virus does not persist for more than 3 and 5 weeks in intestine and brain of sick birds, respectively, while neurological disorders may persist longer. Presumably, some of the cormorants examined may have had residual signs and lesions but have been free of virus (14).

Outbreaks with mortality among cormorants in the United States continued in 1995 and 1997. It was determined that the mortality rate was ~32–64% and recorded only among juveniles under 3 weeks of age. After 4 weeks of age, juveniles left the nest and disseminated the infection (15). Experimental studies with infection of eleven 16-week-old cormorants with the velogenic NDV strain did not cause mortality among them, four cases of ataxia and one case of encephalitis were observed. Birds have been shown to excrete the virus within 28 days of infection. The authors suggested that adult cormorants can serve as a long-distance disseminator of infection during autumn migration (16).

In the 21st century outbreaks of NDV among cormorants were recorded in North America in 2005, 2008, and 2010. Phylogenetic studies have shown their relatedness to each other (3). They were all determined to belong to Genotype V, which diverged from genotype VI in the 1970s (17). In 2009–2011, a large-scale monitoring of cormorants in North America was conducted, 1957 samples from nesting and wintering places were examined. Six velogenic strains were isolated in the nesting sites. Authors provided further evidence that cormorants play an important role in the maintenance and circulation of APMV-1 in the wild of North America. During November 2002, a velogenic NDV strain in cormorant wintering grounds in Florida (USA) was isolated. This strain revealed a 100% deduced amino acid identity with viruses isolated in nesting sites in Minnesota and with the turkey isolate from North Dakota circulated in 1992 (18).

All the authors that described NDV epizootics in North America in 1975–2010 underline that despite over two decades of NDV in cormorants of North America, many aspects of the epidemiology of this disease remained unknown (14). Epizootics of Newcastle disease have occurred every other year on a regular basis but it is not known why not every year.

## Outbreaks of VELOGENIC NDV Among Cormorants in Europe and Asia

The history of the study of NDV among cormorants in Eurasia began with the mass mortality among domestic chickens in Scotland in 1949. Macpherson (6) made observations and found that before that outbreak, local hunters had shot several cormorants for home consumption. Cormorants carcasses and their internal organs were accessible for poultry. Since that moment mass mortality among poultry began in Scotland. Laboratory studies have shown the presence of NDV in samples from chickens. Sixty healthy-looking cormorants were shot to confirm the source of the epizootic. Forty per cent of the shot cormorants had antibodies to NDV and six virus isolations were made.Thus, direct evidence was obtained for the cause of the epizootic by transmission of NDV from cormorants to poultry. Then, during 2000–2009 in the European Union, NDV virulent for chickens have been detected in wild birds, domesticated pigeons, and poultry. Very closely related viruses were obtained from a cormorant in Denmark in 2001 and poultry in Finland, Denmark, and Sweden during 2002–2004. It was supposed that Newcastle Disease outbreaks in Europe originated from a wild bird source from Asia (19, 20).

For the first time in Asia, NDV isolates were isolated directly from cormorants in 2014 at Lake Alakol (8). Juveniles of up to 3 weeks of age were affected and the disease was accompanied with significant mortality. Unfortunately, dead birds were not formally registered by veterinary or other governmental authorities because the areas where the outbreaks occurred were located in a nature reserve where access is limited. The virus was isolated from both adults and juveniles. Then, a year later, in the same nesting area of cormorants in 2016, the velogenic NDV was isolated again (9). In 2014, genotype VII was identified, but in 2016, genotype XIII was revealed. This finding shows the presence of an important NDV focus in the wild nature of Central Asia, which can pose a potential threat to poultry farms in the southern regions of the continent during seasonal migrations of cormorants. The proof of this statement is the outbreak of NDV that close to Lake Alakol around the cormorant nesting grounds in southeastern Kazakhstan and Kyrgyzstan (GenBank: MK423875), where outbreaks with massive mortality of domestic chickens occurred in poultry farms, caused by 99% phylogenetically close NDV strains of sub-genotype VIIb (8, 10). So, the connection between NDV cases in wild and domestic birds become apparent in Central Asia. Cormorants wintering places are Iran, India and other countries of South Asia. Phylogenetic analyses has shown that NDV of genotype VII causing mass mortality in poultry still predominant in Asia. The sub-genotype VIIb is regularly isolated in Iran and the countries of Indian sub-continent, whereas VIId sub-genotype circulate mostly in the Far East (21–23).

As to NDV genotype XIII, ancestral strain was recovered from a cockatoo in India in 1982. Genotype XIII viruses widely circulated in Southern Asia (India and Pakistan) during 2003–2013 despite extensive vaccination efforts (24, 25).

Unlike in North America, no isolates very similar to those from nesting sites have been found in the wintering areas of cormorants in South Asia. Below is a comparative analysis between North American and Asian epizootics.

Similar points:

- Mortality occurred only among juveniles up to 3–4 weeks of age, adults shed the virus but did not get sick.
- NDV outbreaks with mortality were recorded during the nesting period in the summer.
- Besides the cormorants, mortality was observed among sympatric gulls and pelicans (8, 9).
- Highly pathogenic for domestic birds
- Outbreaks do not occur every year, but after two or more years.
- In North America, it was unable to isolate the virus during each outbreak. In Kazakhstan, mortality among juvenile cormorants has been reported at Lake Alakol in 2020, but the virus has not been isolated then.
- There is a specific G110R amino acid substitution in cormorants of North America, but in Asia, in addition to cormorants, it has also been found in gulls, pigeons, and domestic chickens.

Differences:

- In North America, NDV from cormorants belonged to genotype V but in Asia, they belong to genotypes VII and XIII. Interestingly, NDV isolated in 2014 belonged to genotype VII but in 2016, belonged to genotype XIII. Both were found in the same location of Alakol Lake in Asia. Genotype V viruses likely emerged in Central and/or South America in 1970s and subsequently introduced into Europe (26, 27) and therefore, this difference can be considered only geographical.
- NDV was found on cormorant wintering grounds in southern USA. Such findings were not registered in Southern Asia where cormorants from Central Asia winter.

As we can see, there are more points making similar North American and Asian epizootics among cormorants than distinguishing, and we can talk about the same phenomenon on both continents.

## Discussion

Based on the available data, we can see that in Asia, as well as in North America, cormorants pose a significant threat to poultry industry. They pose the greatest threat mainly in nesting places, where massive infection of juveniles occurs, followed by dissemination of the surrounding territories.

However, we cannot exclude the possibility that infected cormorants transfer NDV from northern breeding areas to wintering grounds in Asia. It was shown that the virus in North American wintering grounds continually circulates subclinically among susceptible adult and older juvenile birds, which then further reinitiate the cycle during spring migration (28).

To date, there is no information on the isolation of velogenic strains from cormorants in their wintering areas in South Asia, so it is still impossible to consider them a source of a distant transmitter within the continent. There are only assumptions about an indirect relationship between the viruses of cormorants and poultry, when, during an outbreak of NDV in India, a specific amino acid substitution G110R was identified, which was considered inherent only in cormorants. It should also be noted that in Asia this substitution was found not in all cormorants, but only in one out of 10 studied. An important task is to examine the populations of wintering cormorants in the countries of South Asia for NDV. If found, compare them with strains causing mass mortality among poultry in adjacent territories.

Commercial flocks in developed countries are protected from any contact with domesticated poultry or wild birds or any pet birds. So, only sporadic epizootics occur in Europe and North America. The greatest impact of ND may be on village or backyard chicken production that is widely spread in developing countries of Asia. ND is frequently responsible for devastating losses in village poultry. There are many restraints making the control of ND in village chickens in developing countries extremely difficult or even impossible (29). The introduction of birds from informal or illegal live bird markets poses an important source of penetration of NDV into a flock (30). Thus, the openness of poultry in Asia to external sources of infection provides its vulnerability to the introduction of NDV from wild birds as well.

There are still many questions such as whether cormorants or gulls are the primary reservoir of infection, where is the virus being maintained in nature because outbreaks in cormorants and sympatric gulls occur not every year, why the virus cannot be isolated during every outbreak, cormorants can disseminate the virus within a few weeks and is this sufficient to deliver NDV to the southern wintering grounds? The answers are yet to be found but in any case, cormorants, as a massive species distributed in all corners of the globe, living in huge colonies, is the most important link in the epizootic of velogenic NDV in the wild. An indisputable fact is that cormorants are a species, with the course of evolution, adapted to live with velogenic NDV. Mortality is observed only among young birds and it does not reach 100%, adult birds are not susceptible to the virus, which also increases the likelihood of this species as a reservoir in nature.

## Conclusion

It has been shown that the circulation of velogenic strains among cormorants in North America and Asia is of a very similar nature. Meanwhile, in North America, the circulation of phylogenetically close strains has been established both in nesting and wintering colonies, which are very distant from each other. In Asia, this phenomenon has not yet been identified. In the case of the occurrence of similar cases in Asia, we can talk about the existence of a real possibility of the introduction of velogenic strains from the wild into the domestic flocks.

Unfortunately, the production and management for disease control measures are not sufficient in Asia. Poultry farms are concentrated in close proximity to backyard flocks (31) facilitating the uncontrolled possibilities of viral penetration. An example is an outbreak of NDV in poultry caused by a strain that is 99% close to that of cormorants in the southeast of Kazakhstan. The openness of such poultry farms in South Asia to wild fauna, including cormorants, also poses a significant threat of the introduction of velogenic strains from the outside.

Circulation of virulent viruses in vaccinated birds in commercial farms of Asia and their continuous evolution over time suggest the existence of a high environmental viral load of unknown nature (31). This article proposes to pay closer attention to the cormorants migrating from the North to South Asia as one of the possible links between wild natural strains and those in poultry.

## Author Contributions

All authors listed have made a substantial, direct and intellectual contribution to the work, and approved it for publication.

## Conflict of Interest

The authors declare that the research was conducted in the absence of any commercial or financial relationships that could be construed as a potential conflict of interest.
